# ADDovenom: Thermostable Protein-Based ADDomer Nanoparticles as New Therapeutics for Snakebite Envenoming

**DOI:** 10.3390/toxins15120673

**Published:** 2023-11-28

**Authors:** Stefanie K. Menzies, Raquel Arinto-Garcia, Fernanda Gobbi Amorim, Iara Aimê Cardoso, Camille Abada, Thomas Crasset, Fabien Durbesson, Rebecca J. Edge, Priscila El-Kazzi, Sophie Hall, Damien Redureau, Richard Stenner, Johara Boldrini-França, Huan Sun, António Roldão, Paula M. Alves, Robert A. Harrison, Renaud Vincentelli, Imre Berger, Loïc Quinton, Nicholas R. Casewell, Christiane Schaffitzel

**Affiliations:** 1 Centre for Snakebite Research & Interventions, Liverpool School of Tropical Medicine, Pembroke Place, Liverpool L3 5QA, UK; 2 Centre for Drugs & Diagnostics, Liverpool School of Tropical Medicine, Pembroke Place, Liverpool L3 5QA, UK; 3 iBET, Instituto de Biologia Experimental e Technológica, Apartado 12, 2781-901 Oeiras, Portugal; 4 ITQB NOVA, Instituto de Tecnologia Química e Biológica António Xavier, Universidade Nova de Lisboa, Av. da República, 2780-157 Oeiras, Portugal; 5 Mass Spectrometry Laboratory, MolSys Research Unit, Allée du six Aout 11, Quartier Agora, Liège Université, 4000 Liège, Belgium; 6 Architecture et Fonction des Macromolécules Biologiques, CNRS, Aix-Marseille Université, 13009 Marseille, France; 7 School of Biochemistry, University of Bristol, Biomedical Sciences Building, University Walk, Bristol BS8 1TD, UK; 8 Max Planck Bristol Centre for Minimal Biology, Cantock’s Close, Bristol BS8 1TS, UK

**Keywords:** snakebite, antivenom, venom, biologics, ADDomer

## Abstract

Snakebite envenoming can be a life-threatening medical emergency that requires prompt medical intervention to neutralise the effects of venom toxins. Each year up to 138,000 people die from snakebites and threefold more victims suffer life-altering disabilities. The current treatment of snakebite relies solely on antivenom—polyclonal antibodies isolated from the plasma of hyperimmunised animals—which is associated with numerous deficiencies. The ADDovenom project seeks to deliver a novel snakebite therapy, through the use of an innovative protein-based scaffold as a next-generation antivenom. The ADDomer is a megadalton-sized, thermostable synthetic nanoparticle derived from the adenovirus penton base protein; it has 60 high-avidity binding sites to neutralise venom toxins. Here, we outline our experimental strategies to achieve this goal using state-of-the-art protein engineering, expression technology and mass spectrometry, as well as in vitro and in vivo venom neutralisation assays. We anticipate that the approaches described here will produce antivenom with unparalleled efficacy, safety and affordability.

## 1. Introduction

Snakebite envenoming is a neglected tropical disease that is responsible annually for up to 138,000 deaths and 400,000 disabilities in surviving victims [1], disproportionately affecting the most economically and medically disadvantaged communities of Asia, sub-Saharan Africa and Latin America [2]. The first-choice treatment for snakebite is intravenously delivered antivenom, which is produced by hyperimmunising equines or ovines with subtoxic doses of venom(s) and then purifying immunoglobulins from resulting sera/plasma samples. Consequently, the resulting antivenoms are associated with numerous deficiencies, including limited cross-snake species reactivity, poor dose efficacy and high incidence of adverse reactions [3]. The efficacy of current antivenoms is often restricted to the snake venom(s) used as immunogens in the manufacturing process, due to the significant variation in toxin composition observed between the venoms of different snake species, though venom components can differ even between different individuals of the same species or over the lifetime of a single snake [4]. Snake venoms typically contain mixtures of functionally distinct protein isoforms encoded by relatively few toxin families, and are biochemically diverse secretions that cause variable pathologies in snakebite victims (i.e., haemotoxicity, neurotoxicity and/or cytotoxicity) [1]. Venom toxin variation therefore underpins the restrictive cross-snake species efficacy of antivenom, which in turn translates into the limited geographic utility, reduced economies of scale and poor commercial manufacturing incentives associated with these life-saving therapeutics [5].

Most regions affected by snakebite envenoming are home to several species of medically-important snakes; therefore, most antivenom manufacturers use venom from multiple snake species to produce polyspecific (polyvalent) antivenoms for the target region [6]. This inherently results in a smaller proportion of antibodies in the antivenom directed against the toxins from any one species in comparison to antivenoms raised against only one venom (“monospecific” or “monovalent” antivenom), and therefore much higher doses of polyspecific antivenoms are required to effectively treat envenoming [7]. Further compounding the poor dose efficacy of antivenom is evidence that only 5–36% of immunoglobulins purified from the animal sera/plasma are specific to the venom proteins used for immunisation [8,9,10], and only a proportion of those are likely to be neutralising antibodies. The weak potency of antivenoms, which often require multiple vials to effect a cure, increases the risk of adverse reactions to the large quantity (often grams) of intravenously administered, non-human immunoglobulin, while also resulting in increased costs to the patient. Another consideration regarding the accessibility of antivenoms is cold-chain transport and stable low-temperature storage requirements, which frequently prevents their effective distribution in peripheral rural health centres close to the populations that have the greatest need [11].

Snakebite envenoming was recognised as a priority neglected tropical disease by the World Health Organisation (WHO) in 2017 due to the significant public health problem it presents across most of the rural tropics. A number of novel snakebite treatments are under development: monoclonal human/humanised antibodies (or cocktails thereof), non-antibody toxin-binding proteins or peptides, small molecule drugs and aptamers (for reviews on these therapeutic modalities for snakebite see [12,13,14]). These treatments aim to be better tolerated, more affordable and to possess broader snake species utility than existing antivenoms; however, only two are currently in clinical trials [15,16]. Thus, there remains a strong need to explore alternative biological therapeutics that may provide the next breakthrough in snakebite treatment. Here, we describe the proposed development of ADDomer—a novel protein-based nanoparticle therapeutic for snakebite—in our so named “ADDovenom” project.

## 2. ADDomer—A Versatile Thermostable Nanoparticle

**Figure 1:**
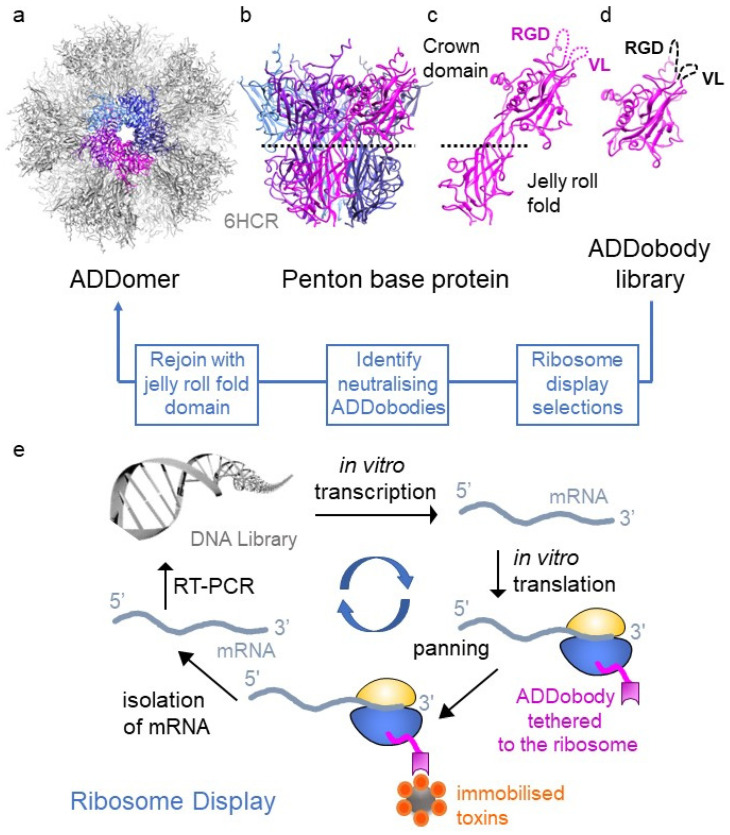
Structure-based design of ADDobody. (**a**) Twelve pentons self-assemble into the ADDomer particle (PDB ID 6HCR [17]). ADDomer is shown in grey, one penton is highlighted in colour. (**b**) The penton consists of five penton base proteins (depicted in magenta, purple, light blue, blue and dark blue). (**c**) Penton base protein comprises a jelly-roll fold multimerisation domain and a crown domain with hypervariable RGD and VL loops. (**d**) ADDobody is the engineered crown domain. RGD and VL loops were randomised in sequence and length to generate the ADDobody library. (**e**) Scheme of ribosome display in vitro selection and evolution. The DNA library encoding the ADDobody library (up to 10^12^ members) is transcribed using T7 RNA polymerase and then translated in vitro. Ribosome-mRNA-ADDobody complexes form and ADDobodies (magenta) can fold outside the ribosomal tunnel due to an unstructured “spacer” fused to the C-terminus of ADDobody. The complexes are used for selection against immobilised antigens/toxins. Non-binders are eliminated during stringent washing steps. mRNA is recovered by dissociating the ribosomal complexes with EDTA, reverse transcribed and PCR amplified (RT-PCR). During PCR, the ribosome-binding site and T7 promoter sequence are reintroduced into the construct. Error-prone PCR introduces mutations into selected binders allowing in vitro evolution. The resulting DNA library is enriched for binders and can be used for a new ribosome display round or cloned into a plasmid to clonally isolate and express selected ADDobodies.

ADDomer is a nanoparticle scaffold derived from the adenovirus penton base protein protomer [17]. In adenovirus, the protomer forms pentons at the vertices of the capsid, providing a base for fibre protein attachment [18]. When expressed recombinantly in isolation, 60 protomers spontaneously self-assemble into pentons that form a dodecahedron, known as ADDomer (for adenovirus-derived dodecamer) (Figure 1a,b). ADDomer-based nanoparticles have been developed as efficient vaccines, e.g., against Chikungunya, SARS-CoV-2 or foot-and-mouth disease [17,19,20,21]. The penton base protein protomer comprises two domains: a jelly-roll fold and a crown domain (Figure 1c). The jelly-roll fold mediates multimerisation into the penton and dodecamer assembly. The crown domain comprises two loops on its surface: the variable loop (VL) and the RGD loop (Figure 1c). The VL and RGD loops are highly variable in sequence and length among the penton base proteins found in different adenoviruses. Immunogenic epitopes and other sequences can be inserted into these loops without compromising efficient folding of the penton base protein and assembly into the ADDomer [17]. The immunogenic epitopes are then displayed on the surface of the ADDomer in 60, 120 or even more copies, depending on the number of epitopes inserted into VL and RGD loops [17].

ADDomer nanoparticles have a number of key advantages that make them uniquely suitable for deployment in developing countries:

1. Thermotolerance up to >45 °C [17,22]. It has been shown that the ADDomer nanoparticle can be stored for one month at ~20 °C; it can be frozen and thawed and/or heated to 45 °C for 1 h, virtually without losing its structural integrity [17]. Consistently, ADDomer does not require cold-chain storage, which constitutes a key logistic advance;
2. ADDomers can be lyophilised [22]. This manufacturing step increases storage life, providing a commercial incentive for manufacturers;
3. ADDomers can be produced as recombinant protein-based nanoparticles with exceptionally good yields using a baculovirus–insect cell expression system, allowing establishment of good manufacturing practices with stringent quality control;
4. Importantly, the penton base protein is the least immunogenic of all the adenovirus capsid proteins. Therefore, ADDomer presents a low immunogenicity scaffold.

The aforementioned characteristics of the ADDomer scaffold—thermotolerance, shelf life, recombinant production and low immunogenicity—are equally desirable for antivenoms. We found that the architecture of the crown domain with its highly variable loops has remote similarities with complementarity determining regions (CDR loops) in antibodies, used to recognise cognate antigens. We hypothesised that the crown domain could be functionalised to bind toxins. Then, the ADDomer could act as a superbinder “sponge” to neutralise toxins. A prerequisite for such a superbinder is the feasibility of a binder molecule representing the crown domain of the protomer. The bipartite architecture of the protomer suggested a possibility to separate the crown from the jelly-roll fold. Thus, we designed ADDobody, comprising only the crown domain and produced the prototype in *Escherichia coli* with excellent yields as a monomeric protein (Figure 1d) [23]. Next, the VL and the RGD loops of the crown domain were randomised in length and sequence, mimicking the CDR loops of antibodies (Figure 1d). The resulting synthetic ADDobody library is used for ribosome display in vitro selections (Figure 1e) against native and recombinantly produced toxins. Selected specific binders are biophysically characterised and tested for toxin neutralisation using enzymatic or cell-based assays. In a next step, the selected toxin-neutralising ADDobody can be rejoined with the jelly-roll fold multimerisation domain to produce the ADDomer-based superbinders [23] (Figure 1). The resulting antitoxin nanoparticle displays 60 binding domains against the toxin target, ensuring high-avidity binding and highly efficient neutralisation of the snake toxin targets.

## 3. The EU-Funded ADDovenom Project

In the ADDovenom project, we will focus on neutralising venoms from two medically-important groups of African snakes that cause severe life-threatening envenoming across the continent, namely saw-scaled vipers (Viperidae: *Echis* spp.) and mambas (Elapidae: *Dendroaspis* spp.) [24]. *Echis* saw-scaled vipers are responsible for the largest numbers of bites and deaths in the northern half of Africa, and their venom mainly causes life-threatening haemorrhage and coagulopathy, as well as debilitating local tissue necrosis [25]. These effects are caused by toxins such as the snake venom metalloproteinases (SVMPs), phospholipases A_2_ (PLA_2_s), serine proteases, C-type lectin-like proteins and disintegrins [26]. Contrastingly, envenoming by *Dendroaspis* mambas causes neuromuscular paralysis, which may become rapidly lethal if respiratory paralysis occurs [24]. Their venom contains several distinct neurotoxins [27], which are mostly members of the three-finger toxin (3FTx) and Kunitz-type toxin families [28,29].

**Figure 2:**
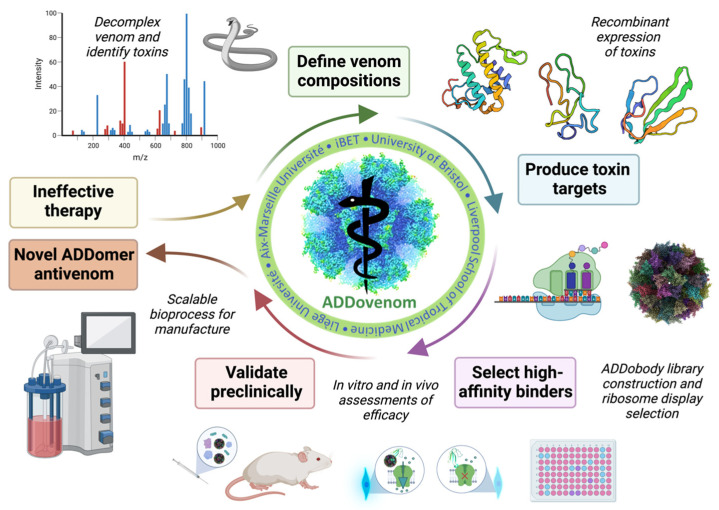
Overview of the ADDovenom project. Mass spectrometry will be used to define the venom composition of nine snake venoms and identify toxins. Toxins will then be produced as recombinant proteins in bacterial and insect cell expression systems. A synthetic library of ADDobodies will be created and screened using ribosome display to identify specific, high-affinity binders to the recombinant toxins. ADDobodies and ADDomers will then be assessed for toxin-neutralising ability using in vitro assays and in vivo models of venom bioactivity. Finally, a GMP-compatible platform will be developed to produce toxin-neutralising ADDomers at scale.

We seek to develop ADDomers and ADDobodies with neutralising capabilities against *Echis* SVMPs, PLA_2_s and disintegrins and mamba 3FTx and Kunitz-type toxins, using the approaches shown in Figure 2. First, venom mass spectrometry will be performed to determine the venom toxin content of each species. Proteomics will identify the protein sequence of priority toxins to be targeted, their post-translational modifications, disulphide bonds and relative abundance in each venom. Priority toxins will then be expressed as recombinant toxins using bacterial and eukaryotic expression systems. A naïve synthetic ADDobody library and recombinant toxins will be used to select for specific, high-affinity toxin binders using ribosome display. The resulting ADDobodies and ADDomers (multimerised ADDobodies) will then be tested for their in vitro and in vivo neutralising capabilities against specific venom toxins and crude venom and will also be assessed in vivo for their safety and pharmacokinetic characteristics. Finally, a good manufacturing practice (GMP)-compatible platform will be developed for the production of toxin-neutralising ADDomers.

### 3.1. Venom Mass Spectrometry and Bioinformatics

One key challenge for the design of toxin-specific therapies is that it is often not apparent which specific isoforms within a family of toxins must be neutralised to prevent mortality and morbidity. The ADDovenom project will take advantage of the versatility of mass spectrometry approaches [30,31] to determine the inventory of peptides and proteins present in the nine African snake venoms collected from saw-scaled vipers (*E. ocellatus*, *E. pyramidum leakeyi*, *E. leucogaster* and *E. coloratus*) and mambas (*D. polylepis*, *D. angusticeps*, *D. viridis*, *D. jamesoni jamesoni* and *D. j. kaimosae*) to be used in this project. From the peptide sequences, the prediction of toxin families and their related pharmacological activities will enable a ranking of toxins according to their toxicological importance to generate a list of “priority” target toxins to be neutralised for each venom. A toxin database containing all this information will be generated to identify the target toxins for recombinant expression (see Section 3.2).

These objectives will be achieved through the application of venomics, a methodology that integrates data generated from transcriptomics and proteomics [32,33]. This rational approach has already been demonstrated to be particularly powerful in exploring the remarkable toxin arsenal of animal venoms. Snake venoms are typically composed of a combination of tens to hundreds of different components, mostly peptides and proteins (>90%), that vary between and within snake species [34,35]. Consequently, identifying the most potent toxins with proteomics can help identify targets for the production of recombinant antivenoms, which present promising new approaches to treating snakebites.

**Figure 3:**
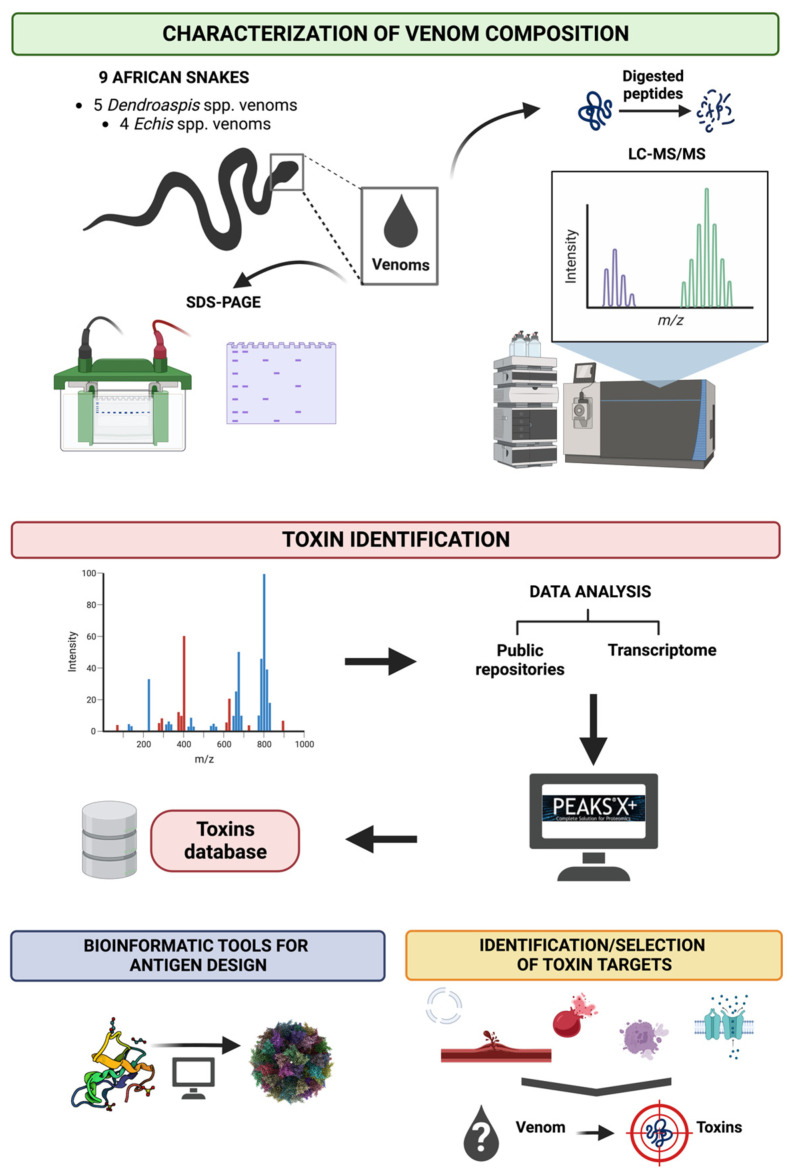
A brief overview to illustrate the aims of the mass spectrometry work package. Nine of the deadliest snakes found in sub-Saharan Africa are selected for this study, including five species of *Dendroaspis* and four species of *Echis*. Shotgun proteomics, along with the use of a database repository and transcriptome data, will be used to generate a toxin database. Bioinformatic tools will then be utilised to predict the biochemical and structural features of the toxins and in combination with predicted/putative biological activity will help to identify and select the most potent toxins in the venom for use in ADDomer production.

In the ADDovenom project, we will characterise the crude venom complexity of nine medically-important African snakes, as summarised in Figure 3. Each venom will be analysed using two-dimensional sodium dodecyl sulphate polyacrylamide gel electrophoresis, leading to a rough evaluation of their structural family. Accurate masses of the most concentrated toxins are obtained by analysing the venoms using matrix-assisted laser desorption/ionization time-of-flight mass spectrometry (MALDI-TOF MS). In addition to this visualisation of venom composition, the results will be exploited to roughly evaluate the abundance of each detected family of toxins, with the aim of extracting information on the pharmacological activity in each venom that requires neutralising. Each venom is then subjected to the bottom-up proteomics strategy; peptides sourced from digested toxins are sequenced by liquid chromatography (LC)-MS/MS using Orbitrap analysers, exploiting their high resolution (70,000 at *m*/*z* 200), mass accuracy (less than 1 ppm at 2 *m*/*z*) and fragmentation efficacy (higher-energy collision dissociation—HCD) for toxin sequencing and identification.

Several methodologies can be considered to increase the efficiency of the sequencing step. The first approach is based on bottom-up shotgun proteomics where each crude venom is chemically treated to reduce and alkylate the cysteines responsible for disulphide bonds formation. The samples are then digested by three enzymes (trypsin, chymotrypsin and GluC serine protease) to amplify the level of information gathered through the MS/MS process, by creating overlapping peptides [36]. The huge amount of proteomics data is then analysed by dedicated bioinformatics tools such as Peaks X (Bioinformatics Solutions) [37]. This software allows the prediction of sequences from MS/MS spectra (de novo sequencing), but also enables matching of protein annotations to the obtained mass spectra/sequences via protein databases extracted from the Uniprot or NCBI servers (e.g., “toxins” AND “snake”). In the case of ADDovenom, the transcriptomics data from mRNA sequencing of the snake venom glands are included to generate a more precise and complete protein database to match with the proteomics data [35,38].

Every toxin sequenced from the nine venoms is added to a home-made database containing the following: toxin name, Uniprot accession number (if known), sequence, mass, toxin family, relative toxin abundance and, where available, predicted/putative biologic activity. By providing the families and the relative abundancies of the toxins, this database constitutes a helpful tool in the process of identification and selection of the most potent toxins to be targeted by the produced ADDobodies/ADDomers. Although the sequence and the abundance does not constitute perfect evidence of toxicity, they serve as an acceptable compromise as a starting point.

In addition, evaluation of what the produced antitoxin molecules are binding to is of prime importance. A methodology allowing the determination at the molecular level of which families of toxins are captured by functionalised ADDomers (qualitative) and how many ADDomers are needed for neutralising a defined quantity of injected venom (quantitative) is desired. Using antivenomic principles [32,39], we will establish a high-throughput methodology based on magnetic beads coated, in the first instance, with antibodies sourced from gold standard antivenoms as a proof of concept, followed by specific ADDomers targeting toxins. After venom incubation, mass spectrometry will be applied to monitor which toxins are captured by the antibodies or ADDomers. This approach will provide rapid and robust evaluation of the potency of antitoxins to selectively and quantitatively bind to targeted toxins. Furthermore, it will provide an evidence base as to whether ADDomer constructs have higher avidities than classical antibody-based antivenoms.

### 3.2. Production of Recombinant Toxins as Antigens for Ribosome Display Selection

Current antivenoms are generated using crude or fractionated venom for immunisation. Recombinant toxin production has the advantage that no venomous snakes are required, tags can be added to facilitate downstream work, and high protein purity can be achieved, allowing the biochemical and biophysical characterisation of the recombinant toxins prior to their use as antigens in ribosome display selection experiments. Ribosome display is an in vitro selection and evolution method ideally suited for generating high-affinity binders from very large synthetic libraries (encoding antibody single-chain variable fragments, nanobodies or engineered proteins) [40,41,42,43,44]. While it is desirable to work with purified, well-characterised toxins, a key challenge is to establish expression protocols for the active toxins; in particular, SVMPs contain many disulphide bridges (some SVMPs comprise 40 cysteines) which make them difficult to express and fold correctly. Successful recombinant protein production, however, will remove the batch-to-batch biological variability in venoms and yield reliably pure protein with defined activity.

Only very few production protocols for toxins are available in the literature [45], and our objective is to develop new protocols, optimised for individual toxins, during this project using prokaryotic (*E. coli*) and eukaryotic (baculovirus–insect cell expression) production systems. Moreover, to test the activity of recombinantly produced toxins, specific functional tests are required for each toxin family, confirming that the purified proteins are correctly folded and bioactive. For instance, the N-terminal and C-terminal residues of venom toxins can contribute to ligand binding and biological activity [46,47]. Therefore, functional tests must be performed to confirm that the addition of tags does not interfere with toxin activity.

In a first approach, all prioritised toxin types (see Section 3.1) will be produced in *E. coli* and purified following a high-throughput protocol that has been successfully validated for a previous EU project called VENOMICS [48] to purify thousands of toxins including 3FTxs, Kunitz-type toxins and disintegrins [48,49]. The toxins are produced in the periplasm of *E. coli* using an N-terminal hexahistidine-tagged redox-active DsbC as fusion tag. DsbC fusions increase solubility and contribute to oxidation of the cysteines, allowing efficient formation of correctly folded disulphide bridges [48]. The fusion protein can be cleaved off using TEV protease after affinity purification, restoring the native N-terminus in the toxin. For ribosome display selections, a non-cleavable C-terminal Avi-tag for biotinylation is fused to the toxins [50]. Importantly, folding of the toxins can be further improved by the co-expression of two cysteine chaperones, protein disulphide isomerase (PDI) and sulfhydryl oxidase Erv1p (the CyDisCo system, see [51]).

Larger toxins with enzymatic activity and composed of several domains, such as SVMPs, contain many disulphide bonds required for structural integrity and possibly also cysteines in the active centre for metal coordination. Due to this increased complexity, bacterial expression systems often fail to produce active enzymes. If the toxins cannot be produced in high yields in *E. coli* or are non-functional, our expression system of choice is MultiBac, a state-of-the-art baculovirus–insect cell expression system [52]. The toxins are then expressed with a melittin signal sequence fused to the N-terminus to ensure that they are secreted into the medium [53] facilitating purification. Insect cell expression may lead to post-translational modification (PTM) of toxins, glycosylation and phosphorylation; the impacts of these PTMs on toxin activity and solubility are largely unknown to date.

A further strategy regarding toxin antigen production for selection experiments we will pursue is generation of “epitope strings” comprising an N-terminal, highly produced fusion protein (e.g., maltose-binding protein) and the most conserved sequences from one toxin family fused via glycine-serine-rich linker sequences [54]—the idea being that conserved regions are functionally important and binding these regions with ADDomers may interfere with their toxicity/function.

Our objective in ADDovenom by implementing these approaches is twofold: to provide the toxin components as antigens for our selection studies and to develop protocols and a knowledge base for how to comprehensively produce toxins of interest in the best suitable expression system.

### 3.3. Evaluation of the Neutralising Ability of ADDobodies and ADDomers

Ribosome display selected ADDobodies and corresponding ADDomers capable of binding recombinant toxins will be assessed for their ability to neutralise toxin function using a panel of serological, phenotypic and functional in vitro assays against crude venom and recombinant toxins. First, serological methods such as end-point ELISA and immunoblots will be used to demonstrate recognition of the target toxin and venom proteins. Typically, an indirect ELISA format is used in which the venom/toxin of interest is coated on an ELISA plate, incubated with the test antibody (i.e., antivenom or other protein therapeutic), and a secondary antibody capable of binding the test antibody/protein used to detect binding. Although ELISAs generally show good correlation with in vivo neutralising ability, for some venoms a poor correlation is observed [55], highlighting the need for rigorous functional in vitro and in vivo testing for each candidate therapeutic beyond serological assays.

**Figure 4:**
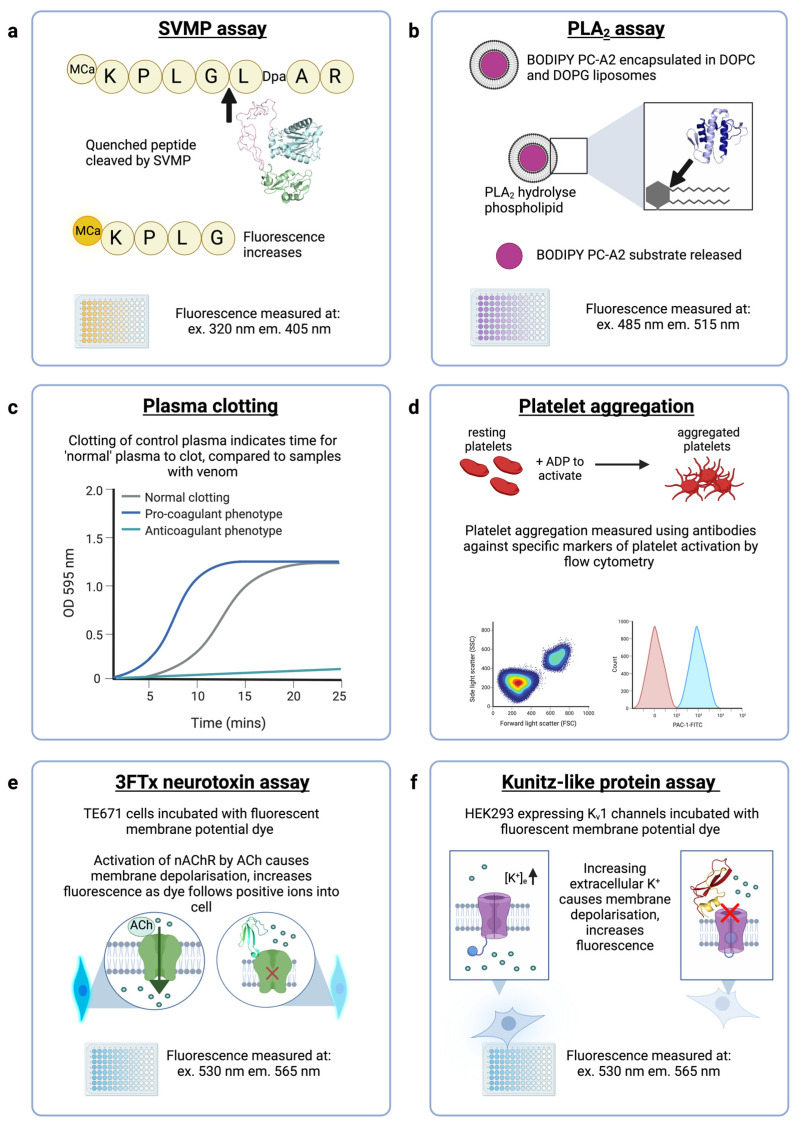
In vitro assays that will be used to measure venom activity and neutralisation by ADDomers. (**a**) The SVMP assay uses a quenched fluorogenic peptide substrate (ES010, BioTechne) which is cleaved by SVMPs, resulting in a free unquenched fragment. Structural model shown (green-pink-cyan) is an *E. ocellatus* SVMP (AlphaFold AF-Q2UXQ5-F1). (**b**) Enzymatically active PLA_2_s are measured using a fluorescent assay (EnzCheck, ThermoFisher) in which a fluorescent substrate (BODIPY PC-A2) is encapsulated in liposomes. PLA_2_s hydrolyse the phospholipids, resulting in increased fluorescence. Structural model shown (blue) is a PLA_2_ from *E. ocellatus* (AlphaFold AF-P59171-F1). (**c**) The plasma clotting assay measures the optical density (OD) of plasma in a spectrophotometer to detect clot formation where a higher OD indicates clotting. A normal plasma clotting control is concurrently run, and compared to venom samples to indicate whether venom is causing pro-coagulant or anti-coagulant effects. (**d**) Flow cytometry assays can demonstrate disturbances in platelet aggregation (indicated by changes to forward and side scatter) and the binding of disintegrins to activated GPIIb/IIIa can be detected with PAC-1-FITC antibody. (**e**) The neurotoxic activity of α-neurotoxins (a subclass of 3FTx) can be measured in a cell-based assay using cells natively expressing muscle-type nAChRs incubated with a fluorescent dye (FLIPR Membrane Potential Assay, Molecular Devices) that measures the change in membrane potential upon acetylcholine-induced nAChR activation. Protein shown (cyan-green) is α-elapitoxin-Dpp2d from *D. polylepis* (PDB ID: 4LFT). (**f**) The neurotoxic activity of Kunitz-type toxins on voltage-gated potassium channels will be measured in a cell-based assay using transfected HEK293 cells expressing human K_v_1.1 or K_v_1.2 channels. Cells are incubated with the fluorescent FLIPR Membrane Potential Assay dye (Molecular Devices) that measures the change in membrane potential upon application of extracellular potassium sulphate which causes cell membrane depolarisation and therefore activation of K_v_ channels. Protein shown (red-yellow) is dendrotoxin-I from *D. polylepis* (PDB ID: 1DEM).

ADDobodies/ADDomers directed against *Echis* SVMP, PLA_2_ and disintegrin toxins will be tested using specific enzyme activity assays and phenotypic assays, as indicated in Figure 4. Neutralisation of SVMP activity will be measured using the fluorogenic assay previously described [56]. Briefly, the quenched fluorescent peptide Mca-KPLGL-Dpa-AR-NH_2_ (ES010, BioTechne) is cleaved by SVMP between the Gly and Leu residues, releasing the fluorescent Mca-containing fragment from the quenching Dpa-containing fragment (Figure 4a), resulting in increased fluorescence. Other assays of SVMP bioactivities, including enzymatic activity, cytotoxicity assays and endothelial cell tube formation assays are reviewed by Macedo and Fox (2016) [57]. The enzymatic activity (phospholipid cleavage) of *Echis* PLA_2_ toxins will be determined using a fluorescent liposome-based PLA_2_ assay (EnzCheck, ThermoFisher) [38]. The assay reagent is prepared by encapsulating a fluorescent dye (BODIPY PC-A2) in a liposome containing dioleoylphosphatidylcholine and dioleoylphosphatidylglycerol (Figure 4b). Liposomes cleaved by PLA_2_ release the fluorescent substrate. Lower enzymatic activity is reported for S49-containing snake venom PLA_2_s which exert cytotoxic activities in a calcium-independent manner [58] which can be measured in standard cell viability assays [59]. *Echis* venoms contain both D49 (enzymatically active) and S49 (enzymatically inactive) PLA_2_ variants.

The haemostatic disturbances caused by *Echis* venoms can be measured using a phenotypic in vitro plasma clotting assay [60], in which the time for “normal” plasma to clot is measured spectrophotometrically, and simultaneously disturbances to plasma clotting (either pro-coagulant or anti-coagulant) by venom toxins are detected by the differences in time to form a clot (Figure 4c). Disintegrin activity can be measured using several different methods that examine the effects of the toxins on platelet aggregation. Traditionally an aggregometer is used [57], but this approach requires specialist equipment and is low throughput. Alternatively, flow cytometry can be used to detect the binding of certain disintegrins (RGD-containing disintegrins and some variations as WGD and KGD) to platelet receptors, and subsequent effects on platelet aggregation can be assessed by flow cytometry comparing forward and side scatter patterns. After platelet activation, the integrin glycoprotein GPIIb-IIIa undergoes conformational changes to expose an epitope that is specifically recognized by the antibody PAC-1 [61]. The binding of PAC-1 antibody is competitively inhibited by RGD-containing peptides, and thus the PAC-1-FITC antibody can be employed in flow cytometry to quantify disintegrin binding to GPIIb-IIIa(Figure 4d). However, some venom disintegrins do not bind the GPIIb-IIIa integrin and therefore require alternative assays for their specific integrin targets, such as fibroblast cell migration assays [62].

ADDobodies/ADDomers targeting the selected mamba toxins will be tested in cell-based assays for antagonism of muscle-type nicotinic acetylcholine receptors (nAChRs) and voltage-gated potassium channels, the targets of α-neurotoxins (a subclass of 3FTx) and Kunitz-type toxins, respectively. To measure the effects of mamba long- and short-chain α-neurotoxins on nAChRs, we will use a functional assay that measures the ability of the receptors to activate in response to binding of the agonist acetylcholine (Figure 4e) [63]. This assay uses a cell line natively expressing muscle-type nAChR to measure the effects of α-neurotoxins, and complements an existing assay that measures the effects of neurotoxins on the neuronal α7-nAChR subtype [64], which are selectively targeted by long-chain 3FTx [65]. Traditional assays of the inhibitory activity of Kunitz-type toxins on voltage-gated potassium channels (K_v_) use electrophysiological recording of *Xenopus oocytes* or mammalian cells engineered to express K_v_ channels [66]. Electrophysiology techniques are considered the gold standard in ion channel research but require specialist equipment and training, and are typically low throughput [67]. In the ADDovenom project, we are developing a higher-throughput (96-well format) assay to measure dendrotoxin-mediated block of K_v_1 channels. HEK293 cells stably expressing tetrameric human K_v_1.1 and 1.2 are stimulated by potassium sulphate, causing a change in membrane potential measured by a commercial membrane potential dye (FLIPR Membrane Potential Assay Kit, Molecular Devices), and antagonism of K_v_ channels by Kunitz-type toxins can therefore also be detected (Figure 4f).

Neutralising ADDobodies/ADDomers will subsequently be tested for venom neutralisation in vivo using murine models of envenoming. We will assess ADDobodies/ADDomers for their efficacy against the lethal systemic effects of *Echis* and *Dendroaspis* venoms using the WHO-approved “effective dose 50” (ED_50_) murine model of snakebite envenoming, where venom challenge and treatment are preincubated together ahead of intravenous co-administration [68]. Evidence of efficacy in this “best case” in vivo model of envenoming will provide a compelling justification to apply more challenging animal models that better recapitulate an envenoming scenario, specifically with therapeutic administration occurring after venom challenge [69].

### 3.4. Scalable Bioprocess for ADDomer Production

**Figure 5:**
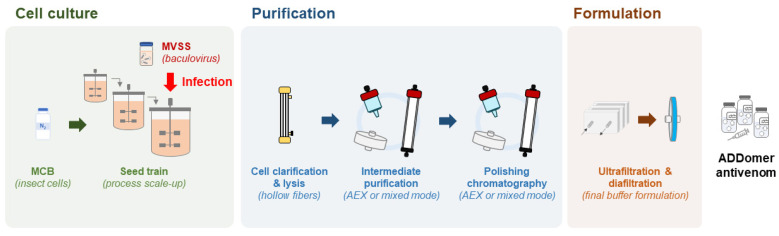
Scalable, GMP-compliant process for ADDomer production. MCB: master cell bank; MVSS: master virus seed stock. See main text for further details.

ADDomer nanoparticles are produced in insect cells, a very competitive manufacturing host, that enables expression at high level of exogenous proteins encoded in a baculovirus genome. As eukaryotes, insect cells can process complex secondary structures, are easy to culture and can grow in serum-free media, require less energy than mammalian cells and have low biosafety requirements [70]. The most frequently used insect cells derive from *Spodoptera frugiperda* and *Trichoplusia ni* and both offer GMP compatible cell lines [71,72,73]. By testing a variety of culture modes and culture medium supplementation strategies, production yields can be increased. Even though the most used culture mode is the batch system, protein-based nanoparticles and other insect cell-derived products can also be produced in fed batch or perfusion [74,75], both to be tested for ADDomer production (Figure 5). A hybrid perfusion process will also be tested, considering the cost and benefit of using larger media volumes and implementing cell-medium separation devices in the production phase [75]. Use of stainless steel or of single-use bioreactors will be considered [76], with future transfer of the final process to a GMP facility in mind, and the latter providing easier technology transfer to GMP. The choice of upstream parameters will be combined with downstream optimisation, ensuring a good compromise between purity and product loss along the process. Once more, ensuring process scalability and GMP compatibility are the main focus. Moving towards a continuous bioprocess has been a major interest as the higher automation will lead to process intensification, reduce steps and shorten the production cycle, thus reducing the production cost [77,78]. Downstream process optimisation encompasses an array of techniques ranging from bench top to industrial scale; however, not all can easily be performed under GMP standards. Centrifugation steps and chromatography with low loading capacity will be avoided (e.g., size exclusion chromatography). The first step will be to optimise the clarification via microfiltration, using for example hollow fibre tangential flow filtration systems. As ADDomer nanoparticles are an intracellular product, this system will be used to separate the cells from the supernatant followed by in situ lysis of cells and recovery of the clarified ADDomer particles (Figure 5). Purification strategies will combine different types of chromatography in bind/elute or flow through modes with anion exchange membranes, these last ones removing DNA, baculovirus and charged host cell proteins. The process will be finalised by ultrafiltration/diafiltration in tangential flow mode and sterile filtration to ensure sterility and stability in the defined storage buffer.

Quality control protocols will be established ensuring reliable efficacy of the antivenom and robust, large-scale ADDomer nanoparticle production. These will cover from the production phase to the final product. During production, virus titre and cell growth rate will be analysed prior to infection. During downstream processing, the intermediate products will be screened by high-performance liquid chromatography (HPLC) for particles quantification and purity analysis. The final product will be screened for the main properties of the ADDomer nanoparticle as an antivenom: size and morphology will be screened by transmission electron microscopy, presence of aggregates will be evaluated using multi-angle dynamic light scattering (MADLS), and HPLC will enable accurate quantification. Impurities will also be controlled by measuring the amount of residual host cell protein, endotoxin levels, host cell DNA concentration and mass spectrometry to identify main contaminants.

For vaccines, cold-chain logistical issues account for up to one third of the final cost and for up to 50% of yearly dose wastage [79,80]. It is likely that similar numbers apply to antivenoms. The need to reduce costs and wastage has driven several studies to develop thermostable, cold-chain free solutions in a variety of systems [81,82,83]. As mentioned in Section 2, ADDomer-based proteins are inherently very stable (Tm > 45 °C) which will translate in a wider range of storage and transport temperatures and consequently reduce the associated cost and reduce reliance on cold-chain logistics. Buffer formulation can play an important role in nanoparticle stability. Even though stability of ADDomer is not a main concern, different buffers (e.g., phosphate-, citrate-based) and stabilisers reported as “generally regarded as safe” (GRAS) will be considered and the cost benefit of their introduction evaluated. Non-animal origin stabilisers such as amino acids and sugars have been reported to increase protein stability by increasing solubility, preventing protein aggregation, or reducing oxidation [84]. ADDomer antivenom final formulation will be tested under different temperature settings as well as freeze–thaw cycles to evaluate long term stability.

## 4. Future Perspective and Conclusions

We anticipate the resulting therapeutics arising from the ADDovenom project will feature numerous advantages over existing antivenoms in terms of efficacy, safety, affordability and manufacturing ability. Existing antivenom production uses animal immunisation with whole venom, which does not take into account the immunogenicity or toxicity of the numerous proteinaceous contents of venom; thus, many of the antibodies raised against venom are directed against non-toxic or low-abundance toxins. Our approach uses mass spectrometry and bioinformatics to rationally identify priority toxins to target. The low immunogenicity of the protein scaffold and the ability to manufacture ADDobodies and ADDomers to GMP-standards in bacterial and insect cells, respectively, will result in a reliable product with improved safety profiles over existing animal-derived antivenoms. The multi-modality format of ADDovenom (i.e., 38 kDa ADDobodies and 3.5 MDa ADDomers) lends itself to providing both a local treatment with characteristics suited to topical application and/or transcutaneous delivery, whilst the high-avidity ADDomers (60 binding sites) will be suited towards neutralising systemic toxins in the circulation. Finally, ADDobodies and ADDomers show impressive thermostability without the need for cold-chain storage, can be lyophilised to extend shelf life and can be produced recombinantly in exceptionally good yields at a competitive production cost. Collectively, these characteristics provide a strong rationale for the ADDovenom project and the future discovery of toxin-specific ADDomers as novel therapeutics for tropical snakebite.
